# Transcriptome Profiling of Wheat Seedlings following Treatment with Ultrahigh Diluted Arsenic Trioxide

**DOI:** 10.1155/2014/851263

**Published:** 2014-11-27

**Authors:** Ilaria Marotti, Lucietta Betti, Valeria Bregola, Sara Bosi, Grazia Trebbi, Giovanni Borghini, Daniele Nani, Giovanni Dinelli

**Affiliations:** ^1^Department of Agricultural Sciences, University of Bologna, Viale Fanin 44, 40127 Bologna, Italy; ^2^Department of Public Health and Infectious Diseases, “La Sapienza” University of Rome, Piazzale Aldo Moro 5, 00185 Rome, Italy; ^3^Italian National Health System, Lombardy District, “Azienda Sanitaria Locale” Milan, Corso Italia 19, 20122 Milan, Italy

## Abstract

Plant systems are useful research tools to address basic questions in homeopathy as they make it possible to overcome some of the drawbacks encountered in clinical trials (placebo effect, ethical issues, duration of the experiment, and high costs). The objective of the present study was to test the hypothesis whether 7-day-old wheat seedlings, grown from seeds either poisoned with a sublethal dose of As_2_O_3_ or unpoisoned, showed different significant gene expression profiles after the application of ultrahigh diluted As_2_O_3_ (beyond Avogadro's limit) compared to water (control). The results provided evidence for a strong gene modulating effect of ultrahigh diluted As_2_O_3_ in seedlings grown from poisoned seeds: a massive reduction of gene expression levels to values comparable to those of the control group was observed for several functional classes of genes. A plausible hypothesis is that ultrahigh diluted As_2_O_3_ treatment induced a reequilibration of those genes that were upregulated during the oxidative stress by bringing the expression levels closer to the basal levels normally occurring in the control plants.

## 1. Introduction

Over the past decades, the growth of public interest and popular use of complementary and alternative medicine (CAM) in the treatment of various health problems have been accompanied by an increased number of research articles and evidence-based approaches assessing efficacy and therapeutic effects. Homeopathy, one of the most widespread and controversial forms of CAM, uses medicines obtained from natural substances (minerals, plants, and animals) subjected to a process of serial dilutions (in different scales) that results in extremely low, often nonmeasurable, levels of active principle.

The central aspect of the dispute concerning the homeopathic treatments is that at very high dilution levels (beyond the Avogadro limit) the probability of the presence of molecules of the original substance is near to zero [1, 2], which, according to conventional scientific thinking, make any biological activity highly unlikely. Despite the fact that there is extensive evidence for ultrahigh dilution (UHD) efficacy both* in vitro* [3–6] and* in vivo* [7–12], the cellular and molecular mechanisms underlying the regulatory processes affected by UHDs must yet be largely elucidated. Various lines of investigation suggest that UHDs might affect some subtle and early levels of signal transduction and/or genetic expression [2]. However, the demonstration of their efficacy in a scientific manner and the exploration of their mode of action within the domain of science have become a challenging field for researchers having to address the many unanswered questions about UHDs by rigorously applying the scientific principles in high quality basic and clinical research [13–15].

One of the most accredited hypotheses to explain the mechanism of action of UHDs* in vivo* [16] is based on the regulation of expression of some specific and relevant genes. The modulatory effects of UHDs have been demonstrated in both prokaryotes and a primitive form of eukaryote like yeast [17, 18]. According to this hypothesis, homeopathic remedies carry specific “signals” or “molecular imprints” of drug that can be identified by specific receptors of the cells as a trigger to turn “on” or “off” some relevant genes initiating a cascade of gene actions to alter and correct the gene expressions that might have gone wrong during production of the pathological disorder/disease condition [16, 19]. Considering that eukaryotic gene expression is an intricate process still not completely understood, it is difficult with the knowledge we have at the moment to precisely suggest the actual molecular mechanisms involved in transmission of information of homeopathic remedies down to the execution level for active recovery process [19]. For validating the basis of UHD efficacy and for understanding their molecular mechanism of action, further development of basic research is highly desirable.

The microarray technology has been proven to be a suitable high throughput tool to simultaneously analyze a large number of genes/targets associated with the therapeutic effects of CAM remedies such as herbal drugs [20]. The working principle for this genomic approach is that the phenotype of a cell, including the function and response to the environment, is ultimately determined by its gene expression profiles. Application of such expression analysis tools may help to understand and characterize the genes and signaling pathways involved in CAM modalities.

Microarray analyses have been used to investigate gene expression variation in human cell lines and animal models after treatment with high dilutions (HDs) and UHDs [21, 22] or homeopathic formulations [23]. Recently, Saha et al. [24] used global microarray profiling of genes to demonstrate that the expression of certain genes in cancer cells treated with UHDs was significantly different from that of the placebo treated cells.

At present, microarray profiling of genes to verify the effects of UHDs on plant systems has never been used. Plant models appear to be useful approaches to address basic questions on homeopathy research as they make it possible to overcome some of the drawbacks encountered in clinical trials, such as placebo effect, ethical issues, duration, and high costs [25–27]. The* in vitro* growth of wheat coleoptiles is one of the first models adopted to investigate the effects of UHDs [28] and has become a classic test system for basic research in this field [29]. In previous experiments [30, 31], we observed a curative effect of arsenic trioxide (As_2_O_3_) at the 45th decimal dilution/dynamization level on wheat seedling growth. This treatment, applied after having poisoned the seeds with a sublethal dose of As_2_O_3_, induced a significant increase in shoot length compared to poisoned seeds grown in distilled water.

In the present study we reproduced our original experiment [30] in order to investigate the global effects of ultrahigh diluted As_2_O_3_ treatments on wheat coleoptiles gene expression through microarray analyses (Gene Chips, Affymetrix, CA). The overall goal was to test the hypothesis whether wheat coleoptiles, grown from As_2_O_3_ poisoned seeds, showed different significant gene expression profiles after the application of ultrahigh diluted As_2_O_3_ compared to water (placebo). At the present state of our knowledge, this is the first time that the microarray profiling approach is being used on plant organisms for gene expression studies on UHDs. This might throw additional insight into the molecular mechanism involved in the biological action of ultrahigh diluted homeopathic drugs.

## 2. Materials and Methods

### 2.1. Plant Material and Experimental Treatments

Seeds of the common wheat (*Triticum aestivum *L.) cultivar “Pandas” (CGS sementi, Italy) were selected for integrity, uniform size, shape, and colour and used to carry out the study.

Part of the wheat seeds was pretreated (poisoned) by immersion for 30 min in a 5 mM As_2_O_3_ watery solution (As_2_O_3_, puriss. p.a. 99.95–100.05%, Sigma-Aldrich, St. Louis, MO, USA; H_2_O p.a. Merck, Darmstadt, Germany), rinsed in tap water for 60 min, dried at ambient air until 12% humidity was attained, and stored in the darkness until use. This sublethal poisoning protocol was selected on the basis of previous range and time exposure-finding trials [30]. Control seeds were pretreated in the same way, but with distilled water instead of As_2_O_3_ 5 mM.

Each seed (poisoned and unpoisoned) was fixed on the upper part of a filter paper (Perfecte 2-extrarapid, Cordenons, Pordenone, Italy) by a piece of clay (0.20 ± 0.05 g). The filter paper was inserted into a transparent polyethylene bag (12 × 20 cm) which in turn was placed into a larger black cardboard envelope covering the roots, but not the shoots [30]. This technique ensured the growth of shoots and roots in natural light and darkness, respectively. 3.2 mL of distilled water or As_2_O_3_ 45x (freshly prepared and supplied by Laboratoires Boiron, Sainte-Foy-lès-Lyon, France) was added in each polyethylene bag during seed growth [30, 31]. As_2_O_3_ 45x treatment (containing a theoretical dose of As_2_O_3_ 1 × 10^−47^ M) was prepared, according to the European Pharmacopoeia, through serial dilutions (in distilled water) at decimal scale (indicated as x) starting from a mother tincture (As_2_O_3_ 0.01 M) and intercalated by mechanical shaking (dynamization process). The distilled water used for control and serial dilutions was from the same batch. The following experimental groups were set up: (1) control (denoted by C + 0): seeds grown in distilled water without prior treatment; (2) treated control (denoted by C + T): seeds grown in As_2_O_3_ 45x without prior treatment; (3) poisoned (denoted by P + 0): seeds pretreated with a sublethal dose of As_2_O_3_ and subsequently grown in distilled water; (4) poisoned-treated (denoted by P + T): seeds pretreated with a sublethal dose of As_2_O_3_ and subsequently grown in As_2_O_3_ 45x. A total of 80 cardboard envelopes (20 per each of the 4 experimental groups) were fixed to a wooden support and seeds were grown in the laboratory at a temperature of 20 ± 1°C in daytime assuring a natural day-night rhythm for 7 days. Treatments (water or As_2_O_3_ 45x) were letter-coded (blind protocol) by a person not involved in the experimentation and were poured into the polyethylene bags according to a completely randomized design. Seven independent replicated experiments were carried out for a total of 140 seedlings grown for each group out of 560 processed seedlings.

The rationale behind poisoning seeds with arsenic and then growing seedlings in ultrahigh diluted arsenic comes from one of the basic tenets of complementary medicines using UHDs, the principle of “like cures like.” In other words, symptoms that are caused by a substance (say As_2_O_3_) can be successfully removed by the application of the same substance at highly diluted microdoses (say As_2_O_3_ 45x).

In our “wheat model” symptoms of illness were induced by treating wheat seeds with a solution of arsenic trioxide. Arsenic is a toxic metalloid widely disseminated in the environment and causes a variety of health and environmental hazards. Due to its widespread occurrence in the environment every organism, from human to bacteria, has mechanism for arsenic detoxification. In plant-based research, arsenic is commonly used for inducing oxidative stress in studies investigating the response of antioxidant defense systems. The right dosage of As_2_O_3_ for the stress was defined in our previous experimentation [30] in which we tested different arsenic aqueous solutions (from 1 mM to 10 mM) for different exposure times (from 30 to 120 minutes). The combination arsenic dilution-exposure time reducing seed germination rate by approximately 15% was chosen as sublethal treatment for imparting a moderate but measurable stress at the system (As_2_O_3_ 5 mM for 30 minutes).

Ultrahigh diluted As_2_O_3_ is generally used in complementary alternative medicine for curing symptoms of arsenic poisoning in patients. We transferred this clinical practice to our plant system and ultrahigh diluted As_2_O_3_ was provided to arsenic intoxicated seeds as treatment for the recovery from the oxidative stress. Particularly, the UHD As_2_O_3_ 45x (containing a theoretical dose of As_2_O_3_ 1 × 10^−47^ M and thus beyond Avogadro's limit) was chosen on the basis of our previous findings showing reproducible and significant stimulating effects on* in vitro* wheat growth [30, 31].

### 2.2. RNA Extraction and Microarray Expression Profiling

Among the seven experimental replications, three of them were chosen for sampling materials for microarray analyses. From each replicate, 10 seedlings were randomly collected within each set (control, treated control, poisoned, and poisoned-treated) and pooled. A total of 3 bulks (biological replicates) per set were obtained and used for RNA extraction and microarray analyses (3 biological replicates per set for a total of 12 hybridizations).

Total RNA extraction was performed on frozen (−80°C) material with the Nucleospin RNA plant kit (Macherey Nagel) according to manufacturer's instructions. RNA concentration was determined spectrophotometrically. RNA quality controls, cRNA preparation, labeling, hybridization, washing, staining, and scanning procedures were performed by Genopolis (Department of Biotechnology and Bioscience, University of Milano-Bicocca, Milan, Italy), as described in the Affymetrix technical manual. Briefly, total RNA integrity was assessed with Agilent Bioanalyzer (Agilent 2100 RNA 6000 Nano LabChip, Agilent Technologies, Palo Alto, CA, USA) and the RNA integrity number (RIN) was calculated. Only high quality RNA preparations, with RIN greater than 7, were used for microarray analysis.

Five micrograms of total RNA for each sample was used in a reverse transcription reaction (One-Cycle Target Labeling Assay, Affymetrix) to generate first-strand cDNA. After second-strand synthesis, double-strand cDNA was used in an* in vitro *transcription reaction to generate biotinylated cRNA. After purification and fragmentation and relative quality control checks by an Agilent Bioanalyzer, the biotinylated cRNA was used for hybridization. Fragmented cRNAs were hybridized to the standard arrays for 16 h at 45°C; the arrays were then washed and stained using the fluidics station and scanned using a GeneChip Scanner 3000 with Affymetrix GCOS software.

Total RNA was processed for use on GeneChip Wheat Genome Array (Affymetrix, Santa Clara, CA, USA) containing 61,127 probe sets which interrogates 55,052 transcripts for all 21 wheat chromosomes in the genome. 59,356 probes sets represent modern hexaploid (A, B, and D genomes) bread wheat (*T. aestivum*) and are derived from the public content of the* T. aestivum *UniGene Build #38 (April 24, 2004). 1,215 probe sets are derived from ESTs of a diploid near relative of the A genome (*T. monococcum*), a further 539 represent ESTs of the tetraploid (A and B genomes) durum wheat species* T. turgidum*, and five are from ESTs of a diploid near relative of the D genome known as* Aegilops tauschii*. Probe sets consisted of pairs of 11 perfect match (PM) and mismatch (MM) 25-mer oligonucleotides designed from the 3′ end of exemplar sequences, with nucleotide 13 as the MM. Array annotation information is available on the NetAffx data analysis center http://www.affymetrix.com.

### 2.3. Affymetrix Chip Data Analysis

Microarray data are available in the ArrayExpress database (http://www.ebi.ac.uk/arrayexpress/) under accession number E-MEXP-3666.

Microarray data analysis was performed with the Affymetrix package encoded in the R language. Affymetrix CEL files were imported using the methods in the Microarray Suite 5.0 (MAS5, the standard Affymetrix algorithm) which involves background correction and normalization across all of the chips in the experiment. To reduce the number of noninformative genes a filter based on an interquartile range was applied (interquantile range, IQR = 0.2).

Gene expression profiles were normalized by dividing the value for a gene for the control value and then calculating the log2 of the ratio. The identification of differentially expressed genes (DEGs) was addressed using a linear modeling approach (Limma) and empirical Bayesian methods [32] together with false discovery rate correction of the *P* value (adjusted *P* value ≤0.05) [33]. The DEGs were further filtered by selecting genes showing a log2 fold change (log2 FC) >1 or <−1.

Annotation analysis of induced and repressed genes was performed using WheatPLEX, a plant ontology database (http://www.plexdb.org/) and HarvEST:WheatChip (http://harvest.ucr.edu/). Putative functions were assigned if the *E*-value was less than 1*e* ^−10^. Alignments with a higher score were visually inspected and annotated if a reasonable degree of homology was observed. Genes were classed as unknown if no reasonable alignments were found.

Pathway analysis was conducted with MapMan 3.5.1 (http://mapman.gabipd.org/web/guest/mapman-version-3.5.1) [34] using the wheat-specific Taes_AFFY_0709 mapping file. Transcripts were mapped into a custom MapMan pathway [35]. The transcript abundance of each gene was scaled to range between −3 and 3, and values were averaged across each BIN.

All individual genes within a functional category were represented as a square box and their expression levels were shown in a colour (red-blue) scale.

### 2.4. Gene Expression Analysis Using Quantitative Real-Time PCR (qRT-PCR)

The reliability of the Affymetrix GeneChip data was confirmed by quantitative RT-PCR (qRT-PCR) analysis of a set of 55 genes chosen on the basis of their involvement in stress-related functional categories (lipid metabolism, histones and cellular structural proteins, stress proteins, cell signaling, transduction and transport proteins, secondary metabolism, protein metabolism, and cell detoxification systems) (Table 1). qRT-PCR experiments were performed using StepOnePlus (Applied Biosystems, UK). RNA from C + 0, C + T, P + 0, and P + T groups from all three biological replications used in the GeneChip experiment was used for validation. Reverse transcription was performed using High-Capacity cDNA Reverse Transcription Kitsaccording to the standard protocol of the supplier (Applied Biosystems, UK).

A pair of primers for each of the selected genes (Table 2) was designed by following a set of stringent criteria, as generally suggested in qRT-PCR protocols (e.g., Primer Express Software v2.0 Application Manual, Applied Biosystems). Target sequences were obtained from the Affymetrix website. Each PCR reaction contained 1x Power SYBR(R) Green PCR Master Mix (Applied Biosystems, UK), 20 ng of cDNA, and 900 nM of forward and reverse primers in a total volume of 20 *μ*L. Reaction conditions were as follows: 95°C for 10 min; 40 cycles of 95°C for 15 s; and 60°C 1 min. To confirm the specificity of amplification, melting curve analysis was carried out after the last cycle of each amplification. All samples were amplified in triplicate from the same RNA preparation and the mean value was considered. For each primer pair, no-template controls were also run. Quantification of the relative changes in gene expression was performed by using the 2^−ΔΔCt^ method [36] with wheat glyceraldehyde-3-phosphate dehydrogenase (GAPDH) used as the endogenous control.

Correlation between results from the array data and qRT-PCR was tested using Pearson correlation coefficient* R* [37].

## 3. Results and Discussion

### 3.1. Microarray Analysis

As the most marked biological effect of As_2_O_3_ 45x was observed in shoot length [30, 31], transcriptome analyses were performed on the coleoptiles of 7-day-old wheat plantlets.

Among a total of 61,127 probe sets contained in the microarrays, 40,398 (66%) produced detectable hybridization signals under our experimental conditions.

To identify genes expressed differentially, the twofold cut-off criterion (log2 fold change >1 and <−1) was adopted [38]. Annotations were assigned to all of the genes that exhibited differential transcript accumulation according to four different groups of comparisons (Table 3): three comparisons (treated control versus control, poisoned-treated versus control, and poisoned-treated versus poisoned) were done to identify genes differentially regulated in response to the application of ultrahigh diluted As_2_O_3_ in both unpoisoned and poisoned systems. A further comparison (poisoned versus control) was investigated to highlight genes involved in seedling growth as a response of arsenic poisoning of seeds.

According to the adopted criteria, a total of 592, 747, 378, and 643 probe sets were identified as differentially regulated in the comparisons between C + T and C + 0, P + 0 and C + 0, P + T and C + 0, and P + T and P + 0, respectively (Table 3).

When comparing the growth in water of poisoned (P + 0) and unpoisoned (C + 0) seeds, it is evident that most of the genes (85%) were upregulated (635 out of 747). By growing poisoned seeds in the presence of As_2_O_3_ 45x (P + T) instead of distilled water, the number of involved probe sets was strongly reduced (378) with 332 up- and 46 downregulated probe sets. It seemed that the treatment of poisoned seeds with ultrahigh diluted As_2_O_3_ induced a normalization of gene expression by bringing it closer to the basal levels usually occurring in the control plantlets (C + 0).

In the unpoisoned system (C + T versus C + 0) a total of 592 probe sets were differentially expressed and among them 71% (421 probes) were downregulated, indicating that the growth of seedlings in the presence of As_2_O_3_ 45x induced a general underexpression of the majority of affected genes. A similar trend was observed in the poisoned system (P + T versus P + 0) where 65% of transcripts showed a downregulation in response to As_2_O_3_ 45x application as compared to water.

Comparison of the affected transcripts between specific interactions was considered in order to differentiate common and/or group specific probe sets.

**Figure 1:**
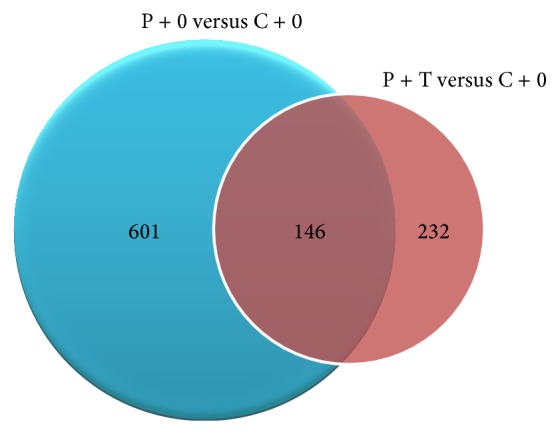
Venn diagrams showing the number of gene transcripts in poisoned (P + 0) and poisoned-treated (P + T) seedlings as compared to the control (C + 0).

**Figure 2:**
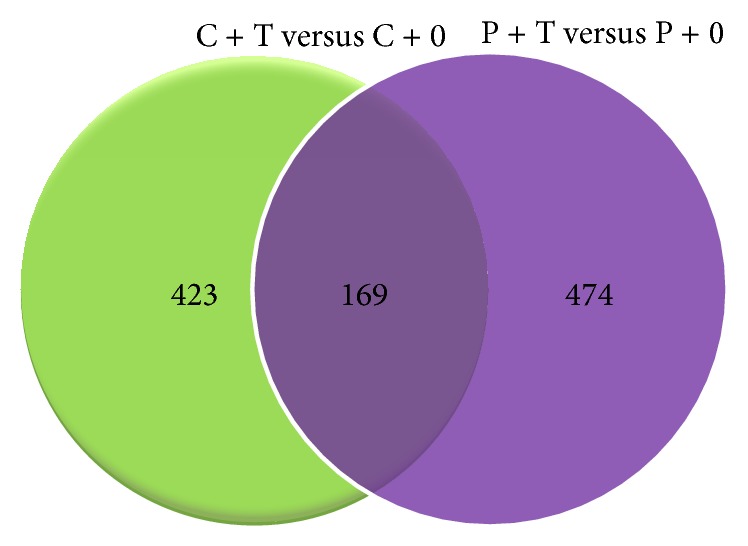
Venn diagrams showing the number of gene transcripts in seedlings grown in ultrahigh diluted As_2_O_3_ as compared to their respective control in both poisoned (P + T versus P + 0) and unpoisoned (C + T versus C + 0) experimental sets.

In the poisoned system, the comparison between P + 0 versus C + 0 and P + T versus C + 0 groups (Figure 1) revealed 146 probe sets common to both interactions, whereas 601 and 232 transcripts were specifically regulated in seedlings grown in water and As_2_O_3_ 45x, respectively. This suggested that the application of ultrahigh diluted arsenic, besides inducing a fewer number of genes, influenced the expression of sets of genes different from those involved in the growth of seedlings in the presence of water. Analogously, by considering the comparison between poisoned and unpoisoned seeds grown with ultrahigh diluted arsenic in relation to their respective controls grown in water (C + T versus C + 0 and P + T versus P + 0), 169 probe sets were common for both interactions, whereas 423 and 474 genes were specifically induced in the C + T versus C + 0 and P + T versus P + 0 interactions, respectively (Figure 2).

The observed gene expression variation induced by UHDs is in line with other literature reports. The regulation of gene expression as mechanism of action of UHDs has long been advocated by Khuda-Bukhsh and coworkers [14, 16, 39, 40] on the basis of various scientific evidences from both the higher organisms like mammals and lower organisms like yeasts and bacteria. According to this gene-regulatory hypothesis, ultrahigh diluted remedies carry specific “signals”/“information” that can be identified by specific receptors of the cells. These “signals” can act as a “trigger” for turning “on” or “off” some relevant genes, initiating a cascade of gene actions to alter and correct the gene expressions that went wrong to produce the disorder/disease. Another recent speculation brings about the hypothesis of an epigenetic action (that affects gene expression rather than gene structure) of UHDs via water-mediated electromagnetic information transfer of specific molecular signals [41]. The ability of potentized homeopathic drugs to cause/modulate histone acetylation/deacetylation activities in some cancer cells has recently been observed [19]. These results strongly vindicate the gene regulatory hypothesis that the potentized homeopathic drugs actually work through regulation of expression of relevant genes, particularly through epigenetic modifications [19].

### 3.2. Pathways

To have a wider picture of the molecular mechanisms occurring at cellular level, the microarray data were investigated for effects on expression of genes belonging to known biochemical pathways or cellular processes in order to gain better insight into the impact of ultrahigh diluted As_2_O_3_ on wheat seedling growth.

**Figure 3:**
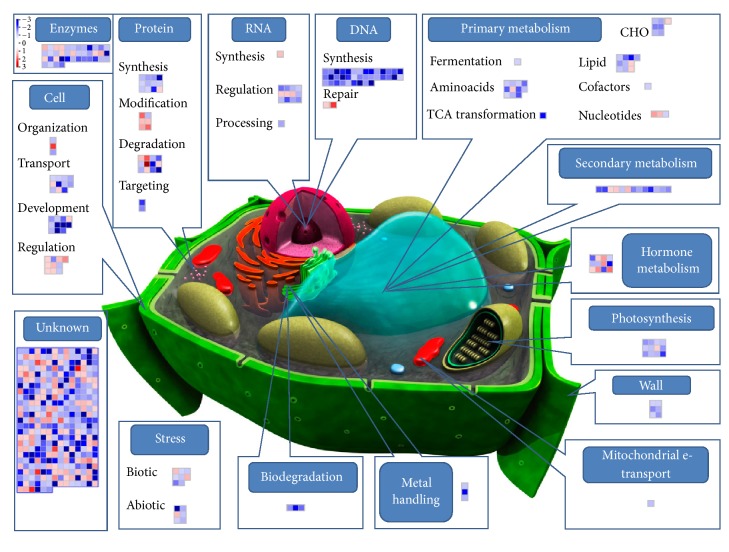
Effect of ultrahigh diluted arsenic on wheat coleoptile gene expression (C + T versus C + 0). Gene expression changes are graphically depicted by MapMan format version 3.5.1 in the frame of a custom-designed cell metabolism overview. Each single square graphically represents one gene identified by microarray analysis and involved in the specific process illustrated. The log2 color scale represents the mean expression ratio (poisoned/control) and ranges from −3 (dark blue), downregulated, to +3 (dark red), upregulated. The complete dataset used for MapMan analysis and related details are given in Table S3 (see Supplementary Material available online at http://dx.doi.org/10.1155/2014/851263).

**Figure 4:**
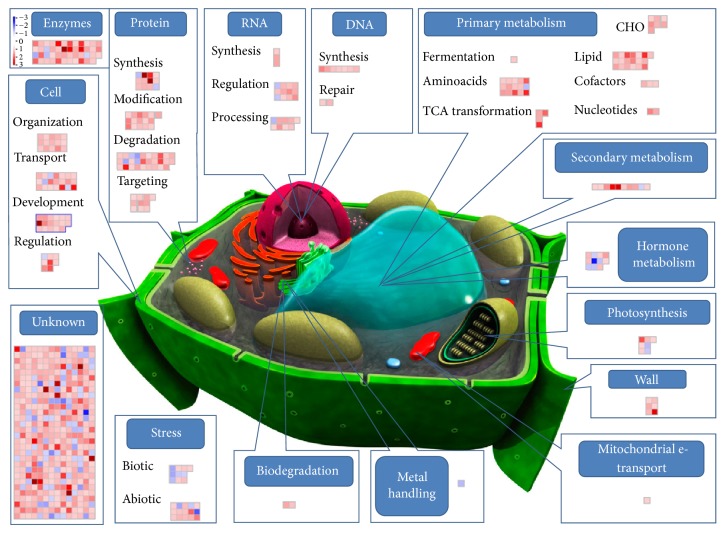
Effect of wheat seed poisoning with a sublethal dose of As_2_O_3_ (5 mM) on coleoptile gene expression (P + 0 versus C + 0). Gene expression changes are as Figure 3. The complete dataset used for MapMan analysis and related details are given in Table S1.

**Figure 5:**
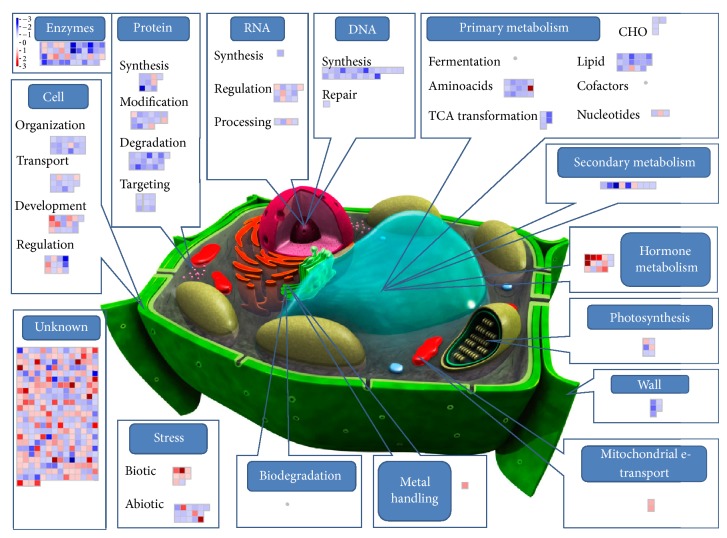
Effect of ultrahigh diluted arsenic on wheat coleoptile gene expression (P + T versus P + 0) grown from seeds poisoned with a sublethal dose of As_2_O_3_ (5 mM). Gene expression changes are as Figure 3. The complete dataset used for MapMan analysis and related details are given in Table S4.

**Figure 6:**
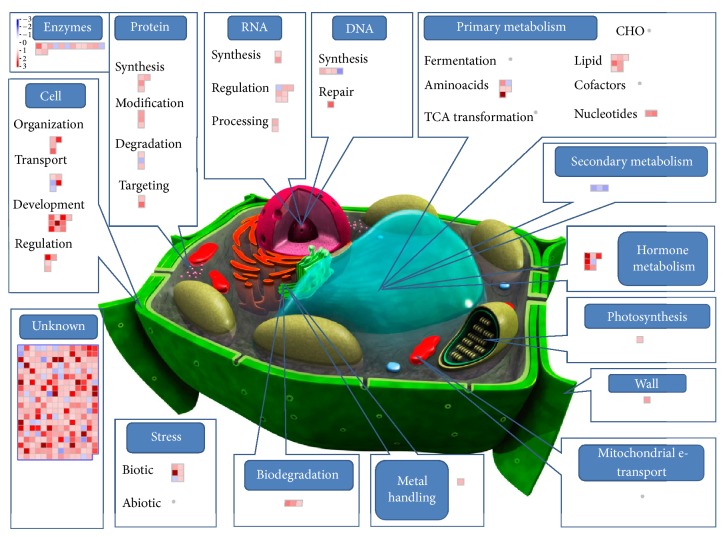
Effect of ultrahigh diluted arsenic on wheat coleoptile gene expression (P + T versus C + 0) grown from poisoned seeds as compared to unpoisoned seeds. The complete dataset used for MapMan analysis and related details are given in Table S2.

To this aim a custom MapMan image pathway was generated and used for visualizing probe sets induced and/or repressed in four pairwise comparisons: treated control (C + T) versus control (C + 0) (Figure 3), poisoned (P + 0) versus control (C + 0) (Figure 4), poisoned-treated (P + T) versus poisoned (P + 0) (Figure 5), and poisoned-treated (P + T) versus control (C + 0) (Figure 6).

Probe sets differentially expressed in the studied groups of comparison were sorted into different functional categories representative of the main cellular processes: genes involved in primary (aminoacid, lipid, nucleotide, fermentation, cofactors and vitamins, tricarboxylic acid transformation, and carbohydrates), secondary, and hormone metabolism, photosynthesis, cell wall, mitochondrial e-transport, metal handling, and xenobiotic biodegradation, genes that encode stress related proteins (biotic and abiotic), genes involved in general cell activities (organization, transport, development, and signaling), genes encoding for enzymes, genes involved in protein metabolism (synthesis, modification, degradation, and targeting), and genes associated with RNA (synthesis, regulation, and processing) and DNA (synthesis, repair) regulation mechanisms.

Most classes of those genes are known to be induced during plant response to environmental stresses and encode for products (i) that directly protect plant cells against stresses such as heat stress proteins (HSPs) or chaperones, late embryogenesis abundant (LEA) proteins, osmoprotectants, detoxification enzymes, and free-radical scavengers such as secondary metabolites; (ii) that are involved in signaling cascades and in transcriptional control, such as kinases, histone proteins, and transcriptional factors; and (iii) that are involved in general metabolic functions (lipid and protein metabolism).

#### 3.2.1. Poisoned (P + 0) and Poisoned-Treated (P + T) versus Control (C + 0) Seedlings

In our study, the effect of seed poisoning with arsenic trioxide was inferred by considering the transcriptome response of poisoned (P + 0) versus control (C + 0) experimental sets. For this comparison a total of 743 data points were represented in the custom MapMan image pathway (Figure 4). The majority of probe sets were consistently upregulated, highlighting the activation of a myriad of biochemical responses aimed at contrasting the effects of seed arsenic exposure. By considering instead poisoned-treated (P + T) versus control (C + 0) plants, the effect of ultrahigh diluted arsenic on seedling transcriptome profile could be hypothesized.

In both groups of comparison, our microarray data suggested the regulation of various genes involved in cell primary metabolism (Tables S1 and S2). In the poisoned group (P + 0) almost all probe sets were upregulated indicating a concerted cell response to arsenic poisoning. Several evidences demonstrate that plant exposure to arsenic does result in the generation of reactive oxygen species (ROS) that can directly damage proteins, amino acids, and nucleic acids and cause membrane lipid peroxidation [42–47]. In our study most of the genes were associated with aminoacid and lipid metabolism. In particular 13 probe sets were involved in aminoacid synthesis pathways and were overexpressed; two other genes were related to tryptophan degradation, with one of them being overexpressed and the other one downregulated. The same diffuse upregulation was observed for genes involved in lipid metabolism. These included 19 probe sets encoding for enzymes undergoing the synthesis of fatty acids and phospholipids as well as lipid degradation enzymes such as lipases. Other genes involved in nucleotide synthesis, carbohydrate (CHO) metabolism, tricarboxylic acid (TCA) transformation, cofactor and vitamin metabolism, and fermentation pathways were also positively induced in the P + 0 group.

Conversely, in the P + T group only the expression of few genes resulted to be affected and particularly those involved in aminoacid metabolism, fatty acid, and phospholipid and nucleotide synthesis. Fatty acids and phospholipids are crucial components of cellular membranes, suberin, and cutin waxes that provide structural barriers to the environment. They contribute to inducible stress resistance through the remodeling of membrane fluidity and the release, through lipase activity, of *α*-linolenic acid and as modulators of plant defense gene expression [48]. Modification of membrane fluidity is thus an important functional response of plants during general abiotic stress.

Genes belonging to the subclasses fermentation, TCA-transformation, CHO, cofactors, and vitamins did not appear in the map of the P + T group indicating that their expression was comparable with that of the control group.

A differential regulation of genes encoding for stress-related proteins involved in the response to biotic and abiotic stimuli was also observed. Most of the genes encoding for various heat-shock, cold and drought/salt responsive proteins were consistently upregulated in the poisoned group as compared to the control. Those same genes were not affected in the poisoned-treated group where the treatment with As_2_O_3_ 45x seemed to bring the levels of gene expression close to those of the control. The synthesis of such proteins has been reported to increase after various forms of abiotic stress [49]. These molecular chaperones function by helping in the folding of nascent polypeptide chains, the refolding of denatured proteins, and the prevention of irreversible protein aggregation and insolubilization [50]. They increase the rate of folding and thus increase the resistance of cells under stress conditions.

Other stress-related proteins are those involved in biotic agents recognition. This recognition activates signal transduction cascades that lead to the activation of plant defense mechanisms. In our study we observed the modulation of genes encoding disease-related proteins that were both up- and downregulated in the P + 0. In the P + T group the probe sets encoding for proteins related to biotic stress were mostly upregulated. The activation of pathogen-related pathways could sound senseless as the stressor applied in our wheat model is of abiotic origin; however, a growing body of evidence supports the notion that plant signaling pathways consist of elaborate networks with frequent crosstalk, thereby allowing plants to regulate both abiotic stress tolerance and disease resistance [51].

Among the negative effects on plants induced by arsenic, a reduction of the photosynthesis rate was observed by other authors [52]. Arsenic damaged the chloroplast membrane and disorganized the membrane structure, inducing functional changes of the integral photosynthetic process. The treatment of seeds with As_2_O_3_ in our wheat model (P + 0) turned into an upregulation of some of the genes encoding for proteins of the photosystems, ATP synthase, and enzymes of the Calvin cycle. Beside one probe set encoding for photosystem polypeptides, all those genes resulted to be not induced in the P + T group as compared to the control. This suggested that in seedlings poisoned and then grown in the presence of ultrahigh diluted arsenic the photosynthetic processes occurred similar to those of the control plants (C + 0).

The effects of arsenic-poisoning were also evident on genes involved in nucleic acid synthesis, regulation, and processing. Seven different histones (the chromatin structural proteins) together with two DNA repair factors were upregulated (log2 fold change >1.1) in the P + 0 group. Fewer histone genes were involved in the response of the P + T group, and among them one was even downregulated as compared to the control plants. The same trend was observed for RNA where, against a diffuse upregulation of genes involved in transcription, regulation of transcription, and processing observed (for a total of 24 probe sets) in P + 0 plants, only few genes involved mainly in RNA transcription regulation were positively modulated in the P + T group.

Histone modifications are among the epigenetic mechanisms regulating gene expression in response to developmental and environmental stimuli [53]. Since epigenetic modifications do not affect the DNA sequence, these changes show the potential of plasticity and underlie environmental influences. Recent works demonstrated that UHDs emit electromagnetic signals [54], indicating the suggestive hypothesis of an epigenetic action of UHDs on biological systems [41]. The ability of ultrahigh diluted remedies to trigger epigenetic modifications, like methylation/demethylation of DNA and acetylation/deacetylation of histone, has also been recently demonstrated in some cancer cells [55].

The involvement of genes encoding for metal binding proteins and factors related to xenobiotic biodegradation were also observed in poisoned seedlings after As_2_O_3_ 45x treatment (P + T group). As those genes were slightly induced or even negatively modulated in the poisoned group, a specific upregulation, induced by ultrahigh diluted arsenic, of metal-handling targeted defense mechanisms could be hypothesized.

As regards cell activities, various genes encoding proteins involved in organization, transport, development, and signaling mechanisms exhibited altered expression levels in response to seed poisoning with As_2_O_3_. Among cell transport systems, ATP-binding cassette (ABC) transporters mediate the translocation of a wide range of structurally unrelated molecules across biological membranes [56, 57]. In our study, ABC transporters and multidrug resistance proteins were specifically activated in the poisoned group. Those kinds of transporters were not present in the P + T group indicating that they were expressed at the levels found in the control plants (C + 0). As regards signaling molecules, protein kinases, which were upregulated in seedlings of the poisoned group (P + 0), are known to play crucial roles in signal transduction pathways in all eukaryotes. Some kinase genes were also overexpressed in seedlings grown with As_2_O_3_ 45x, suggesting the importance of those signal proteins in response to environmental stimuli. Literature reports suggested that the oxidative tissue damage induced by heavy metals [58, 59] could result also in the elevation of Ca^2+^ concentration in plant cells, in which Ca^2+^ played an important role in free radical scavenger induction [60, 61].

Three probe sets encoding for calcium-related transport and signaling systems were activated in response to arsenic poisoning of seeds (P + 0), whereas in P + T those probe sets were not affected, suggesting again a reequilibration of gene expression, induced by As_2_O_3_ 45x, to the levels of the control (C + 0).

Among cell signaling molecules, hormone-related proteins are known to have a key role in the pathways that govern biotic and abiotic stress responses [51]. Our results showed the involvement of some genes encoding for proteins responsive to auxins, gibberellins, ethylene, jasmonate, and abscisic acid that resulted to be moderately overexpressed (log2 FC from 1.0 to 1.3) in the poisoned group (P + 0). The same trend was observed in the P + T group where a more marked upregulation (log2 FC from 1.3 to 2.8) of those hormone-related proteins was observed. According to those observations a strengthening effect in the hormone-mediated defense response of poisoned seedlings may be attributed to the As_2_O_3_ treatment. Other hormone-related genes (mainly responsive to brassinosteroids) were significantly downregulated (log2 FC from −1.2 to −2.3) in both P + 0 and P + T groups. Brassinosteroids have a critical role in plant growth regulation, including stem elongation [62]. Their negative modulation in poisoned and poisoned-treated seedlings as compared to control is consistent with shorter coleoptile lengths recorded in the biological observations.

Another important functional class generally involved in plant response to biotic and abiotic stresses is secondary metabolites. Genes involved in the metabolism of isoprenoids, phenylpropanoids, phenols, and wax were consistently induced (log2 FC from 1.0 to 2.6) in the poisoned group. This is in line with other findings reporting the stress-protective role of those metabolites in mitigating arsenic toxic effects [63]. Conversely, only three probe sets involved in flavonoid metabolism were differentially expressed in the P + T group and all of them resulted to be downregulated as compared to the control (C + 0).

The observed downregulation, along with the fact that genes induced in the poisoned group were not present in the poisoned-treated group, may suggest a putative effect of ultrahigh diluted As_2_O_3_ in bringing the metabolic situation of poisoned seedlings close to that of the control plants.

Proteolysis plays an important role in stress response pathways. One important function is the removal of damaged proteins to avoid accumulation as potentially harmful aggregates and to eliminate proteins with compromised activity [64].

On the whole we observed the upregulation of many genes encoding for proteases, peptidases, proteasome, and ubiquitin systems in the response of P + 0 plants following the application of an oxidative stress (sublethal dose of arsenic). This upregulation was not confirmed for most of the genes in the P + T group where gene expression was comparable or even downregulated as compared to the control group.

Other genes involved in protein metabolism such as synthesis, posttranslational modification, and targeting were found to be markedly upregulated in the poisoned group. As observed for protein degradation, only a limited number of genes belonging to the above listed functional subclasses were activated in the poisoned-treated group, whereas all other probe sets had expression levels close to those observed in the control.

Gene expression analysis revealed that, among cell detoxification systems, most cytochrome P450 enzymes, peroxidases, and glutathione transport proteins were strictly regulated in the response of the plant to several abiotic stressors such as UV damage, heavy metal toxicity, mechanical injury, drought, high salinity, and low temperatures [65].

In our study most of the genes encoding for cytochrome P450 proteins were strongly upregulated (from 1.1 to 3.4 log2 fold changes) in the P + 0 group, whereas those same genes were not induced in poisoned seeds grown with As_2_O_3_ 45x (P + T), indicating that expression values were comparable with those of the control group (C + 0).

Intuitively, response to conditions associated with oxidative stress must, in part, rely on endogenous antioxidative defense mechanisms required to maintain cellular homeostasis. Glutathione-S transferases (GSTs) are ubiquitous proteins in plants that play important roles in stress tolerance and detoxification metabolism. Detoxification of xenobiotics is considered to be the main function of plant GSTs, but other functions include protecting cells from a wide range of biotic and abiotic stressors, including pathogen attack, heavy metal toxins, oxidative stress, and UV radiation [66–68]. However, in our study only tree probes encoding for GSTs were involved, one in P + 0 group (underexpressed) and two in the P + T group (one downregulated and one upregulated). This observation clearly contradicts our expectation about a diffuse GST induction in response to the imposed oxidative stress. Considering that our experimental end-point was at 7-day after treatment, it is probable that changes in GST transcript abundance did occur only within the first hours after stress application as observed by other authors [69, 70].

Peroxidases are a group of enzymes located in vacuole, cell wall, thylakoid membranes, and extracellular space that are involved in plant defense against reactive oxygen species.

Our microarray data revealed a significant underexpression for one or more probe sets in both P + 0 and P + T groups as compared to the control. This downregulation of peroxidases was also observed by Chakrabarty et al. [71] reporting the effects of arsenate and arsenite stresses on rice seedlings. According to their observations, peroxidases downregulation was correlated with the activation of a chloroplast-targeted lipoxygenase that presumably caused a damage to thylakoid membranes. In our study, along with the downregulation of peroxidases, the activation of cell wall degradation systems was also observed, suggesting an arsenic-induced damage of cell wall components.

#### 3.2.2. Treated Control (C + T) versus Control (C + 0) and Poisoned-Treated (P + T) versus Poisoned (P + 0) Seedlings

If we consider the two interactions “treated control (C + T) vs control (C + 0)” and “poisoned treated (P + T) vs poisoned (P + 0)”, a clear effect of ultra-high diluted As_2_O_3_ in two distinct systems (poisoned and unpoisoned) can be observed. From Figures 3 and 6 the occurrence of an overall reduction of gene expression of most of the involved probe sets can be deduced. Literature evidences demonstrated that elicitors can induce a downregulation of the transcript levels of several genes concomitant with the induction of a defense response in plants. Therefore, the downregulation of gene expression can be also associated with defense responses [72].

In the context of this massive gene underexpression, some gene categories resulted to be upregulated in both systems (unpoisoned and poisoned) (Tables S3 and S4). It is the case of genes grouped under the functional category “hormone metabolism”: in the C + T versus C + 0 group the upregulation involved genes responsive to auxins, brassinosteroids, and jasmonate, whereas in the P + T versus P + 0 group a consistent overexpression of abscisic acid and ethylene-regulated genes was observed. This suggested that in some way ultrahigh diluted As_2_O_3_ activated hormone-mediated response pathways, independently of whether the systems had been poisoned or not. A similar behavior was observed for some genes involved in the phenylpropanoid pathway, particularly for probe sets encoding for the enzyme phenylalanine ammonia lyase (PAL). The activation of stress-responsive hormones and secondary metabolism has been already observed for several plant species where stress hormones like jasmonic acid or ethylene resulted to mediate elicitor-induced accumulation of secondary metabolites [73]. It is presumable that ultrahigh diluted As_2_O_3_ acted like a sort of elicitor that induced a plant response in terms of selective activation of specific pathways (hormone-mediated and secondary metabolites) along with a general downregulation of most other genes.

Other upregulations were observed, even if not shared by both systems. In the C + T versus C + 0 group various probe sets encoding for protein kinases were activated, along with other genes involved in protein modification and degradation. Protein interaction and/or modification is the best way for the cell to strictly regulate the activity of transcription factors in signal transduction pathways that require rapid gene induction [74]. It is thus highly probable that ultrahigh diluted As_2_O_3_ represented a kind of signal for the cell in the unpoisoned system.

In the P + T versus P + 0 group, the upregulation concerned genes involved in metal handling, mitochondrial electron transport, and biotic stress, whose induction was presumably part of the plant recovery mechanism in response to the stress imposed at the system (seed poisoned with As_2_O_3_). The observation that ultrahigh diluted As_2_O_3_ induced a response in the unpoisoned seedlings (C + T versus C + 0) comparable with the trend observed in the poisoned system (P + T versus P + 0) suggested that the artificial growth of wheat did impart a sort of stress to the system, indicating that* in vitro *conditions are by no means the perfect substitutes for the natural ones.

The results of the present study clearly demonstrated that the expression profiles of several classes of genes in plants treated with ultrahigh diluted As_2_O_3_ were significantly different from that of the placebo (water) treated plants. The present microarray data in experimental wheat genome lend further critical support to the gene regulatory hypothesis first proposed by Khuda-Bukhsh [14, 16]. Several reports by Khuda-Bukhsh and coworkers revealed quite clearly that certain ultrahigh diluted homeopathic remedies were capable of altering gene expression while the placebos invariably failed to do so; this became more evident with the results on the ability of potentized homeopathic drugs to trigger key epigenetic events like methylation/demethylation of DNA and acetylation/deacetylation of histone [55], which are an integral part of the epigenetic modification route [19]. However, how a homeopathic remedy diluted beyond Avogadro's limit can elicit response in a cell and bind with the receptor is not yet precisely known. Some recent lines of evidence suggest that certain changes in physical-chemical parameters of the solvent occur when homeopathic drugs are potentized by the procedure of successions and serial dilutions [19], and nanoparticles of the same drug are still retained in the diluent with their medicinal property preserved [19, 75]. How this translates into changes in biological systems is presently at a speculative stage and needs to be further explored [19].

The possible pathways and sites of action of UHDs have also been discussed by Khuda-Bukhsh and coworkers [24, 76–79]. Presumably, their action is mediated through cytokine responses. Thus, administration of UHDs can elicit response in suitable signal proteins and can either upregulate or downregulate such signal proteins to bring back the recovery of the patient to normal health [42].

### 3.3. Gene Expression Analysis Using qRT-PCR

Real-time PCR is generally considered the “gold-standard” assay for measuring gene expression and is often used to confirm findings from microarray data. A total of 55 probe sets encoding for genes involved in stress-related functional categories (lipid metabolism, histones and cellular structural proteins, stress proteins, cell signaling, transduction and transport proteins, secondary metabolism, protein metabolism, and cell detoxification systems) and that resulted to be over- or underexpressed in the four investigated groups were randomly selected for real-time PCR validation.

The gene expression profiles determined by microarray and real-time PCR were found to be well correlated *P* < 0.05 (*R* ^2^ = 0.77). This indicated that microarray data were reliable. The correlation coefficient of 0.77 is very good considering that microarray data are semiquantitative and subject to error for multigene families where different transcripts could hybridize to similar probes on the array. qRT-PCR data are more specific since the amplification of single transcripts is confirmed by melting curves.

## 4. Conclusions

In this study, the analysis of the transcriptome profile of wheat coleoptiles grown from arsenic poisoned seeds suggested the involvement of a complex network of regulatory pathways in As-response. Along with genes harboring general metabolic functions, the changes in expression profiles of many known genes involved in or affected by general abiotic stresses were observed.

The effect of ultrahigh diluted As_2_O_3_ was particularly striking on the poisoned system: a reduction of gene expression levels to values comparable to those of the control (water-treated) was observed for several functional classes of genes. It was the case of probe sets involved in cytochrome P450, histones, stress-related protein synthesis, and other important functional classes implicated in cell response to environmental stimuli. This suggested complex and concerted actions of the cell involving most metabolic functions from genome rearrangement to proteome and metabolome modification resulting in final cell survival.

The understanding of how these genes are connected with each other and how they are linked to biological responses must be further investigated. A plausible hypothesis is that As_2_O_3_ 45x treatment induced a reequilibration of those genes that were upregulated in the poisoned group by bringing their gene expression levels closer to the basal levels usually occurring in the control plants. We cannot surely affirm whether this response is advantageous or not for the plant, but the biological measurements of plant shoots and roots [30, 31] seemed to confirm a vigorous growth of As 45x-treated seedlings as compared to water-treated seedlings.

The present work should be considered as the first step of a science-based route towards the comprehension of UHD remedial effects. So far UHD possible mechanisms of action remain intangible theories, and it will be important ultimately to substantiate these with compelling research evidence.

The findings of the present study contributed to support the gene regulatory hypothesis first proposed by Khuda-Bukhsh [16]. However, the actual molecular mechanism involved in transmission of information of the UHD down to the execution level has to be more clearly ascertained [19]. In our opinion, further investigations integrating the genomic, proteomic, and metabolomic approaches could shed light onto the molecular responses triggered by UHDs not only in plants but also in humans.

## Supplementary Material

Complete dataset of up- and down-regulated gene sequences, resulting from MapMan analysis, in the four pairwise comparisons. Table S1: poisoned versus control (P+0 vs C+0); Table S2: poisoned-treated versus control (P+T vs C+0); Table S3: treated control versus control (C+T vs C+0); Table S4: poisoned-treated versus poisoned (P+T vs P+0).

## Figures and Tables

**Table 1:** List of the 55 genes, selected for microarray validation by qRT-PCR, grouped on the basis of their involvement in stress-related functional categories.

Functional categories	Gene ID
Lipid metabolism	BG909536; BJ270709; CK198840; CK207205; CK208220
Histones and cellular structural proteins	BQ838979; CA635455; CA660860; BE416616; BJ251672; BQ607338; CA628396; CD868502; CD869995; CA719316; BJ225202; BJ262694; BJ274465; BJ306445; BJ308450; BJ320258; BQ294672; BQ168973
Stress proteins	BJ322693; CA613417; CA699507; CA708152; CA708660; CK205521
Cell signaling, transduction, and transport proteins	BF484849; BJ226504; BJ246114; BJ303391; BJ312841; BQ578355; CA629836; CA640553; CA681335; CA744534; CA747224; CD453390; CK203324
Secondary metabolism	CK210556
Protein metabolism	BJ228692; BJ243882; BJ256146; CA599873; CA700404; CA731980
Cell detoxification systems	CA642191; CA676257; CA676553; CA685752; CA719198; CK198851

**Table 2:** Gene ID and respective forward and reverse primer nucleotide sequences (5′–3′) used for amplification by qRT-PCR. Functional categories of the genes are detailed in Table 1.

Gene ID	Primer sequence 5′→3′
Wheat GAPDH	TTGCTCTGAACGACCATTTC
	GACACCATCCACATTTATTCTTC
BG909536	TGCCTGGCGCTTGCA
	CCAGTACGAGGACCAAATCGA
BJ270709	TGCGGCTGGTTTCAAGTCA
	CCCGGACGAGAGAAAACAAC
CK198840	GGCCGGCTTACGCATGT
	GGCCGTGGAGCTTACCATAA
CK207205	CATGGTCGGTTGCCATCAG
	TTCCCCAGTCAAGCTTTGGT
CK208220	GTAGCTGTCGAAGAACACATGCTT
	GCTCTGCGACACGAGGTACA
BQ838979	GCCCGTACGTGCAGTACAAA
	CGTAGGCCCGCATCTTGTAG
CA635455	CCCGGCCTGGGAAGAA
	TGGCTATGGCTTGGTTAGCA
CA660860	CCTGCTCGTCTCCCAGGAT
	GCGCAGGCATCGTTTCTT
BE416616	TGCTCAAGATCTTCCTCGAGAAC
	GGCGTGCTCGGTGTAGGT
BJ251672	GCAAGACGCTTCCTCTTCGT
	GTTGAAGCTTGTTCTTTTGCTTTG
BQ607338	CTCCTCCACCTCGATCCAAA
	CATGGCTGACGGCTTCCTT
CA628396	AAGGGCTTGTTCGGCTAATTC
	CAAACCAATCTTTGCCAATCC
CD868502	TCCTCTTAGCCTGATGTGTTCATC
	CGACCACGACACGAACAAAC
CD869995	TCGCGACACCGCTTAGGT
	CAGAATCCAACCAACCAAAGCT
CA719316	CATCTTTAGCCATCCTCACAGAAA
	TCTGTCAGATTCGTCGAGCAA
BJ225202	GGCCGTCACCAAGTTCACA
	CCGCCATCAATCCATGAAA
BJ262694	TTGAAGTCCTGCGCGATCT
	AAGAGCACGGAGCTGCTGAT
BJ274465	TCACCCATGCTCGACGAA
	TGAGATCCTGTATGTAATGCCTGCTA
BJ306445	GGATCCCATCTTAGTCCATGAGA
	GAGTAGCCGTCGTCCCCATA
BJ308450	GCGCAGGACACAGAGTGAAA
	CGGTGTACTGATGGTTCCAGTTT
BJ320258	CAAGCTTGAGCACTCTCTGGAA
	TCGTGTGCCTAGTGTCTGTGTTAA
BQ294672	CATAGCCAGCGCACTAGATTCA
	GGCTGACCCTGCTGTTTCC
BQ168973	CCAGGGCAACACGAGCTT
	TGCCAAACCCAATCCTAAGG
BJ322693	GCCACCAGGAGAGCAAACAG
	CGCTGCGGGTGATGAACT
CA613417	TGCCCGTTCTGCAGCAA
	CGTCCCGTGTCCACTGTCT
CA699507	CGGCACCACCTACTCATGTG
	CCATGGTCATTGGCGATAATC
CA708152	CCAGAAGCAGCGCAACGT
	GCGGTGCTTTTAGCCTTTAGG
CA708660	AAGCACCAAAAGAAGGCACAA
	TCTTGGTGTCATGGGAGAAATTT
CK205521	GCCATCCCCGGTTCTTG
	GCTGCCGATCGCTTATGTC
BF484849	ACAGGTAACTGGGAGGGCAAT
	AGATCTGAATTATGTGATGGGCATT
BJ226504	CTTCACCCTGCCCAAGGA
	CCGCTGTTGCTGATCACAAA
BJ246114	GGCAGGCACACGAACGA
	GCGAGGGCTCTAGGTTACAACA
BJ303391	GCTGAAGAACCCGGAATCG
	TCCTCCTGGAATGCTGCAA
BJ312841	CGCATGACAGGAGAGCGTTT
	TGTCCCACTGACAGCAACAGTT
BQ578355	CCATCGCCTTCTACATCAAGGT
	GCGACGAGCTTCTCGATCTC
CA629836	TTGCACACCCTTCGACAAAG
	TTTGCTGCTTGCTCTTGAAAAA
CA640553	AACCCATGCGTCACCTGATC
	CCTACCTTCCATGAGCTCAACTG
CA681335	CTGGGAGTGCTGGTTGCAT
	TGGCATGATAGGCTTCCTTGA
CA744534	CACGGATCTGGGACCTGAA
	CCAGCGTGCCCTTCTAGCT
CA747224	CAGAGCAAACCCAACTCGAATA
	CTTCTAGCCGCCATTTGGAA
CD453390	TCCGCGCTTGTTCAAGTACTC
	TTGGGTGCTGCCTTCCA
CK203324	TCTGAGCCCGGCACCAT
	TGACGTTCCTGCCGATGTC
CK210556	AGAAGATGCGTGCGGTTCTC
	GTTCGGCCTCACCATTGG
BJ228692	GGCCTTCTTGGCCATCCTTA
	CTTGCCCGGGAGATAAATCA
BJ243882	CAGGAAGCAGATCGCTGTTG
	AGGATGGCTACGTTCTGGTTTC
BJ256146	GGATAGGGCAGCTGCTTTCA
	GGCTTGACAGCCACCTTAGG
CA599873	CATGAGGACACCATGAGCAAA
	ACCAGCATCCAGAATACCAGAAG
CA700404	ACTACCGCCTCCCCAACAC
	CCGCGACTCGAAGAAGAAGT
CA731980	AGCGGCAGCTACGTGAACTT
	TGGGCTCCTCCACCATGA
CA642191	GGTAGAGAGGATCGCTGAAATCA
	GCCGCCAGCTTAGGAACTT
CA676257	TGGCAAACGCCGAGTACA
	GCGCCCAGTTGAAGCAGTA
CA676553	TTACCCCGCCAAAAAATGTATT
	ACGGCTGCCTGCACATCT
CA685752	GCTGGAGAGACCGTTTTGTCTT
	CGGTTTCGGCCTGTGATTTA
CA719198	CACCGCCAGAGGGTCTTTC
	CGCACTGCTCGATGATATGC
CK198851	GCCGGGCCCTTACGAAT
	CCTTCAAGGTGGCAACAGGTT

**Table 3:** Number of upregulated, downregulated, and total regulated probe sets of the four investigated comparisons.

Comparison	Upregulated	Downregulated	Total
C + T versus C + 0	171	421	592
P + 0 versus C + 0	633	114	747
P + T versus C + 0	332	46	378
P + T versus P + 0	224	419	643

C + 0 = control; C + T = treated control; P + 0 = poisoned; P + T = poisoned-treated.
